# Critical Appraisal of Bivalirudin versus Heparin for Percutaneous Coronary Intervention: A Meta-Analysis of Randomized Trials

**DOI:** 10.1371/journal.pone.0127832

**Published:** 2015-05-26

**Authors:** Anthony A. Bavry, Islam Y. Elgendy, Ahmed Mahmoud, Manoj P. Jadhav, Tianyao Huo

**Affiliations:** 1 North Florida/South Georgia Veterans Health System, Gainesville, Florida, United States of America; 2 Department of Medicine, University of Florida, Gainesville, Florida, United States of America; Harvard Medical School, UNITED STATES

## Abstract

Percutaneous coronary intervention with bivalirudin plus bail-out glycoprotein IIb/IIIa inhibitors has been shown to be as effective as unfractionated heparin plus routine glycoprotein IIb/IIIa inhibitors in preventing cardiac ischemic events, but with a lower bleeding risk. It is unknown whether bivalirudin would have the same beneficial effects if compared with heparin when the use of glycoprotein IIb/IIIa inhibitors was similar between treatment arms. We searched the MEDLINE, Web of Science, and Cochrane databases from inception until March 2015 for randomized trials that compared bivalirudin to heparin in patients undergoing percutaneous coronary intervention. We required that the intended use of glycoprotein IIb/IIIa inhibitors was similar between the study groups. Summary estimates were principally constructed by the Peto method. Fifteen trials met our inclusion criteria, which yielded 25,824 patients. Bivalirudin versus heparin was associated with an increased hazard of stent thrombosis (odds ratio [OR] 1.49, 95% confidence interval [CI] 1.15-1.92, *P* = .002, I^2^ = 16.9%), with a similar hazard of myocardial infarction (OR 1.09, 95% CI 0.98-1.22, *P* = .11, I^2^ = 35.8%), all-cause mortality (OR 0.88, 95% CI 0.72-1.08, *P* = .21, I^2^ = 31.5%) and major adverse cardiac events (OR 1.04, 95% CI 0.94-1.14, *P* = .46, I^2^ = 53.9%). Bivalirudin was associated with a reduced hazard of major bleeding (OR 0.80, 95% CI 0.70-0.92, *P* = .001, I^2^ = 63.5%). The dose of heparin in the control arm modified this association; when the dose of unfractionated heparin in the control arm was ≥ 100 units/kg, bivalirudin was associated with a reduction in major bleeding (OR 0.55, 95% CI 0.45-0.68, *P* < .0001), but when the dose of unfractionated heparin was ≤ 75 units/kg, bivalirudin was not associated with reduction in bleeding (OR 1.09, 95% CI 0.91-1.31, *P* = .36). Among patients undergoing PCI, bivalirudin was associated with an increased hazard of stent thrombosis. Bivalirudin may be associated with a reduced hazard of major bleeding; however, this benefit was no longer apparent when compared with a dose of unfractionated heparin ≤ 75 units/kg.

## Introduction

Unfractionated heparin has been widely used for anticoagulation during percutaneous coronary intervention (PCI). The addition of glycoprotein IIb/IIIa inhibitors to unfractionated heparin has been shown to reduce peri-procedural ischemic events compared with heparin alone; however, this approach can increase bleeding risk [1].

The Randomized Evaluation in PCI Linking Angiomax to Reduced Clinical Events (REPLACE)-2 trial demonstrated that bivalirudin, a direct thrombin inhibitor, was non-inferior to unfractionated heparin combined with a routine glycoprotein IIb/IIIa inhibitor in preventing major adverse cardiac events (MACE), but with a lower risk of bleeding [2]. Both unfractionated heparin and bivalirudin are approved by the European Medicines Agency and United States Food and Drug Administration and endorsed by the European Society of Cardiology and American College of Cardiology/American Heart Association as acceptable anticoagulants during PCI [3,4].

A recent meta-analysis compared a bivalirudin-based regimen with a heparin-based regimen during PCI [5]. The study concluded that bivalirudin increased the risk of MACE, myocardial infarction, and stent thrombosis. There was significant heterogeneity in major bleeding and bivalirudin was only associated with a reduction in major bleeding when compared with heparin plus a routine glycoprotein IIb/IIIa inhibitor. This is not a novel finding since the reduction in major bleeding with bivalirudin has been consistently observed in analyses in which the control arm routinely used glycoprotein IIb/IIIa inhibitors in addition to heparin [6]. As the routine use of glycoprotein IIb/IIIa inhibitors during PCI is no longer contemporary, and may confound any associations between bivalirudin and ischemic/bleeding events, we aimed to conduct a comprehensive meta-analysis to compare the efficacy and safety of bivalirudin versus heparin during PCI, while controlling for the use of glycoprotein IIb/IIIa inhibitors.

## Materials and Methods

### Data Sources

**Fig 1:**
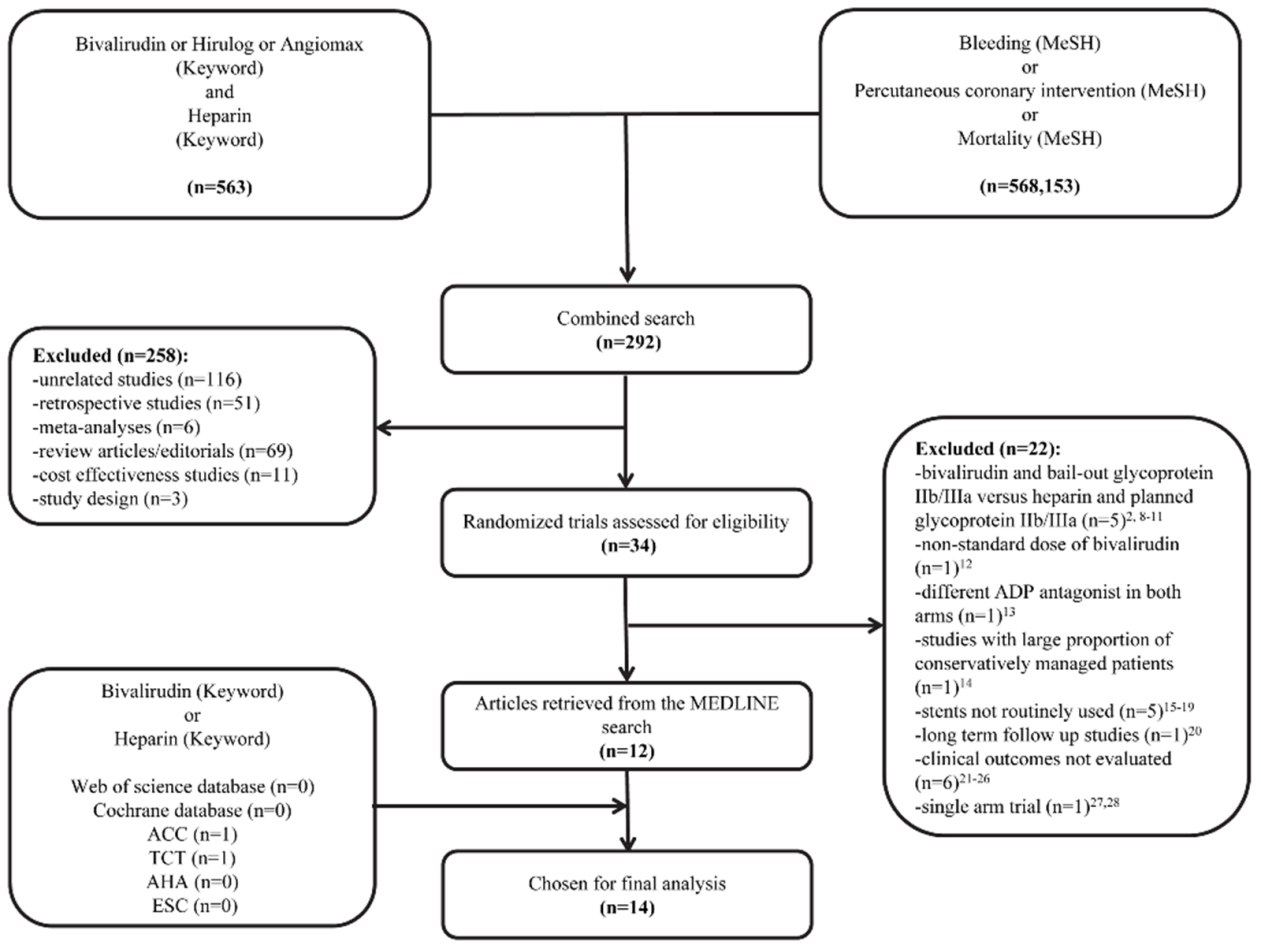
Study selection flow diagram. Summary of how the systematic search was conducted and eligible studies were identified. ACC = American College of Cardiology; ADP = adenosine diphosphate; AHA = American Heart Association; ESC = European Society of Cardiology; GP IIb/IIIa = glycoprotein IIb/IIIa; MeSH = Medical Subject Headings; TCT = Transcatheter Cardiovascular Therapeutics.

We performed a computerized literature search of the MEDLINE database without language restriction from inception until March 2015 using the search strategy shown in Fig 1 [2,7–43]. We also searched both the Web of Science and Cochrane databases using the keywords “bivalirudin” and “heparin”, which did not identify additional studies beyond MEDLINE. Additionally, we searched for abstracts of scientific sessions reported in *European Heart Journal*, *Circulation*, and *Journal of the American College of Cardiology* from 2012 onwards using the same keywords. To ensure that no potentially important studies were missed, the reference lists from the retrieved articles and prior meta-analyses were also checked.

### Selection Criteria

We selected studies that reported clinical outcomes at 30 days (or during hospitalization if 30-day outcomes were not available) in which patients were randomized to receive either bivalirudin or heparin during PCI. We required that patients were randomized to 1) bivalirudin plus a bail-out glycoprotein IIb/IIIa inhibitor versus heparin plus a bail-out glycoprotein IIb/IIIa inhibitor or 2) bivalirudin plus a routine glycoprotein IIb/IIIa inhibitor versus heparin plus a routine glycoprotein IIb/IIIa inhibitor. Bivalirudin was given as a bolus (0.75 mg/kg), followed by infusion (1.75 mg/kg/hour for the duration of the procedure). Heparin could be administered as either unfractionated or low-molecular-weight heparin. The dose of unfractionated heparin ranged from 60 to 140 units/kg. We excluded studies that randomized patients to bivalirudin plus a bail-out glycoprotein IIb/IIIa inhibitor versus heparin plus a routine glycoprotein IIb/IIIa inhibitor, and studies that used different adenosine diphosphate (ADP) receptor antagonists between treatment arms. Additionally, in order to focus on contemporary practice, we excluded trials that did not routinely use stents.

### Data Extraction

Two authors (IYE and AM) independently extracted data on study design, sample size, and other study characteristics from the included randomized-controlled trials using a standardized form. A third author (MPJ) verified the data. Any discrepancies were resolved by consensus of the authors. When necessary for data or article clarification, personal communication was made with select study authors. For all clinical outcomes, we tabulated the number of events that occurred for each outcome of interest in each arm of each trial.

### Outcomes and Definitions

The efficacy outcomes that were tested were stent thrombosis, MACE, all-cause mortality, non-fatal myocardial infarction (MI), and revascularization, while major bleeding, minor bleeding, and net adverse clinical outcomes (NACE) were assessed as safety outcomes.

MACE and NACE were variably defined according to the individual trials (S1 Table) [29–43]. We defined stent thrombosis as definite or probable according to the Academic Research Consortium [44]. Revascularization was defined as urgent, unplanned, or ischemia-driven. MI was defined either as a peri-procedural rise in cardiac biomarkers > 3 times the 99th percentile of the upper reference limit or the combination of ischemic symptoms and/or electrocardiographic changes suggestive of ischemia along with a 3-fold increase in the pre-procedural biomarker level. Most of the trials used the REPLACE-2 criteria for major bleeding (ie, intracranial, intra-ocular, or retroperitoneal hemorrhage, clinically overt blood loss resulting in a decrease in hemoglobin of more than 3 g/dl, any decrease in hemoglobin of more than 4 g/dl, or transfusion of two or more units of packed red cells or whole blood) [2].

### Statistical Analysis

We analyzed outcomes by the intention-to-treat analysis. Summary estimates were principally constructed by a fixed-effect model. We used the Peto method for construction of a fixed-effect summary odds ratio (OR) [45,46]. Additionally, we conducted a Shuster, Guo, and Skyler analysis, which is a random effects method for low—event-rate binomial data [47]. The Cochrane Handbook advises against using inverse variance random effects methods such as DerSimonian-Laird for low—event-rate data [45]. To quantify the statistical heterogeneity for each outcome of interest, we used the I^2^ statistic. I^2^ statistic values < 25%, 25% to 50%, > 50% were considered as low, moderate, and high degree of heterogeneity, respectively [48]. We assessed the risk for publication bias using Harbord’s method [49]. We conducted this analysis according to the Preferred Reporting Items for Systematic reviews and Meta-Analyses (PRISMA) guidelines [50]. Furthermore, we assessed the quality of the trials based on the adequate description of treatment allocation, blinded outcome assessment, and description of losses to follow-up [51]. All p-values were 2-tailed, with statistical significance set at 0.05, and confidence intervals (CIs) were calculated at the 95% level for the overall estimates effect. All analyses were performed using STATA software version 11 (STATA Corporation; College Station, Texas) or SAS 9.3 (SAS Institute Inc.; Cary, North Carolina) according to the applicable method.

### Additional Analyses

In addition to the primary analysis that involved all of the retrieved trials that compared bivalirudin to heparin, we conducted a sensitivity analysis that excluded 1) trials that allowed for upstream use of non-study anticoagulation prior to randomization, and 2) trials that had a modest difference in glycoprotein IIb/IIIa inhibitor use between treatment arms. We also conducted subgroup analyses to explore for possible effect modification: 1) acute coronary syndrome versus elective cases on stent thrombosis and major bleeding, 2) unfractionated heparin (≤ 75 units/kg) versus unfractionated heparin (≥ 100 units/kg) on major bleeding, and 3) majority radial versus majority femoral procedures on major bleeding.

## Results

### Baseline Characteristics

Overall, we identified 15 studies with 25,824 patients available for analysis: 13,255 in the bivalirudin arm and 12,569 in the heparin arm [29–43]. For ACUITY we used the pre-specified cohort of patients that underwent PCI [42], and for EUROMAX we used the bivalirudin arm and the pre-specified cohort of patients that received heparin plus a bail-out glycoprotein IIb/IIIa inhibitor [34]. For the MATRIX trial, glycoprotein IIb/IIIa inhibitor use (i.e., routine or bail-out) was left to operator discretion; however, the majority of patients in the control arm received heparin (100 units/kg) plus a bail-out glycoprotein [29]. The dose of bivalirudin used during the procedure was similar in all of the studies, except for one study in which the bivalirudin dose was 0.1 mg/kg bolus followed by 0.25mg/kg/hour, with an additional 0.5 mg/kg bolus prior to PCI and the infusion increased to 1.75 mg/kg/hour for the duration of the procedure [42]. In EUROMAX, the protocol specified that bivalirudin be continued for at least 4 hours at a dose of 0.25 mg/kg/hour [34]. In MATRIX, 48% underwent a prolonged infusion of bivalirudin after PCI [29]. The mean duration of follow-up was 28.5 days. The mean age was 64 years in the bivalirudin group versus 65 years in the heparin group. The baseline characteristics and follow-up duration are summarized in Table 1. Table 2 reports the medications used in the trials. S2 Table provides measures of study quality [29–43].

**Table 1:** Baseline Characteristics and Follow-up Duration.

Trial (ref#)	Year	Patients, n	Age, mean (SD)	Men, %	DM, %	Prior MI, %	Radial access, %	Follow-up duration	ACT target value, sec	Indication for PCI
**Bivalirudin plus a bail-out glycoprotein IIb/IIIa inhibitor versus heparin plus a bail-out glycoprotein IIb/IIIa inhibitor:**										
**MATRIX [29]**	2015	3,610/3,603	65(12)/65(12)	76/77	NR	NR	50/50	30-days	NR	STEMI/NSTEMI
**BRIGHT [30]**	2015	735/729	57(12)/58(12)	83/82	23/19	4.4/4.5	78/79	30-days	250–300*	STEMI/NSTEMI
**NAPLES III [31]**	2015	418/419	78(4)/78(4)	50/56	45/43	42/38	0.5/0.5	In-hospital	250	Elective
**ACRIPAB [32]**	2014	50/50	68(11)/65(13)	78/60	84/90	40/34	90/78	In-hospital	250	Elective and some ACS†
**HEAT-PPCI [33]**	2014	905/907	63(NR)/64(NR)	72/73	13/15	14/10	80/82	28-days	200	STEMI
**EUROMAX [34]**	2014	1,089/460	61(NR)/62(NR)	75/77	12/17	7/10	48/41	30-days	None	STEMI
**Xiang et al. [35]**	2013	110/108	57(6)/59(5)	92/89	NR	42/42	24/27	30-days	225	Elective
**SWITCH III [36]**	2012	51/49	63(12)/62(13)	73/63	14/20	NR	69/67	In-hospital	200*	Urgent for ACS
**ARMYDA-7 BIVALVE [37]**	2012	198/203	70(8)/70(10)	71/72	67/59	37/34	2/2	30-days	NR	Elective‡
**ARNO [38]**	2010	425/425	69(11)/69(11)	77/75	21/22	41/38	2/2	30-days	250–300*	Elective
**ISAR-REACT 3 [39]**	2008	2,289/2,281	67(10)/67(10)	76/77	27/28	32/30	0/0	30-days	None	Elective
**Bivalirudin plus a routine glycoprotein IIb/IIIa inhibitors versus heparin plus a routine glycoprotein IIb/IIIa inhibitors:**										
**Desphande et al. [40]**	2012	49/52	55(10)/57(10)	90/85	37/42	25/35	NR	30-days	200–250*	Elective§
**TENACITY [41]**	2011	185/198	NR	NR	NR	NR	NR	30-days	225	Mainly urgent for ACS
**ACUITY-PCI [42]**	2007	2,609/2,561	62(NR)/63(NR)	74/73	27/28	30/30	NR	30-days	200–250*	Urgent for ACS
**REPLACE-1 [43]**	2004	532/524	64(12)/64(11)	69/71	31/29	39/45	3/3	In-hospital	200–300*	Elective

* ACT was checked only in the heparin arm

^†^ 32% of patients had non-ST-elevation myocardial infarction

^‡^ Elective in patients with high bleeding risk

^§^ Elective in patients with high ischemic risk

Data are formatted as bivalirudin arm/ heparin arm

ACS = acute coronary syndrome; ACT = activated clotting time; DM = diabetes mellitus; MI = myocardial infarction; NSTEMI = Non-ST-elevation myocardial infarction; NR = not reported; PCI = percutaneous coronary intervention; SD = standard deviation; STEMI = ST-elevation myocardial infarction.

**Table 2:** Study Medications.

Trial (ref#)	ASA, %	ADP-antagonist, %	Clopidogrel, %	Prasugrel, %	Ticagrelor, %	Glycoprotein IIb/IIIa inhibitor, %	Upstream anti-coagulation	Unfractionated heparin dose (units/kg)
**Bivalirudin plus a bail-out glycoprotein IIb/IIIa inhibitor versus heparin plus a bail-out glycoprotein IIb/IIIa inhibitor:** 47/45								
**MATRIX [29]**	NR	83/81	100/100	36/37*	36/37*	4.6/25.8	33% received heparin	100
**BRIGHT [30]**	100/100	100/100	100/100	0/0	0/0	4/6	None	100
**NAPLES III [31]**	100/100	100/100	100/100	NR	NR	0.5/1.3	None	70
**ACRIPAB [32]**	100/100	100/100	12/10	0/0	0/0	0/0	None	60
**HEAT-PPCI [33]**	99/100	99/99	50/50	27/28	61/63	14/16	None	70
**EUROMAX [34]**	100/100	98/98	100/100	31/44	19/6	8/25	None	100^†^
**Xiang et al. [35]**	100/100	100/100	100/100	0/0	0/0	0.9/3.7	None	130
**SWITCH III [36]**	100/100	100/100	100/100	0/0	0/0	4/12	Fondaparinux within 24 hours prior to PCI	60
**ARMYDA-7 BIVALVE [37]**	100/100	100/100	100/100	0/0	0/0	12/14	None	75
**ARNO [38]**	100/100	100/100	100/100	0/0	0/0	15/28	None	100
**ISAR-REACT 3 [39]**	100/100	100/100	100/100	0/0	0/0	0.2/0.2	None	140
**Bivalirudin plus a routine glycoprotein IIb/IIIa inhibitor versus heparin plus a routine glycoprotein IIb/IIIa inhibitor:** NR								
**Desphande et al. [40]**	NR	NR	100/100	NR	NR	100/100	None	70
**TENACITY [41]**	100/100	100/100	68/68	0/0	0/0	100/100	NR	50
**ACUITY-PCI [42]**	98/98	68/68	55/57	0/0	0/0	97/97	Up to 2 doses of heparin	60*
**REPLACE-1 [43]**	100/100	55/57		0/0	0/0	71/73	None	60–70

* Prasugrel and ticagrelor combined together

^†^ Enoxaparin 1 mg/kg twice daily could be used instead of unfractionated heparin

Data are formatted as bivalirudin arm/ unfractionated heparin arm

ADP = adenosine diphosphate; ASA = aspirin; NR = not reported; PCI = percutaneous coronary intervention

### Stent Thrombosis

**Fig 2:**
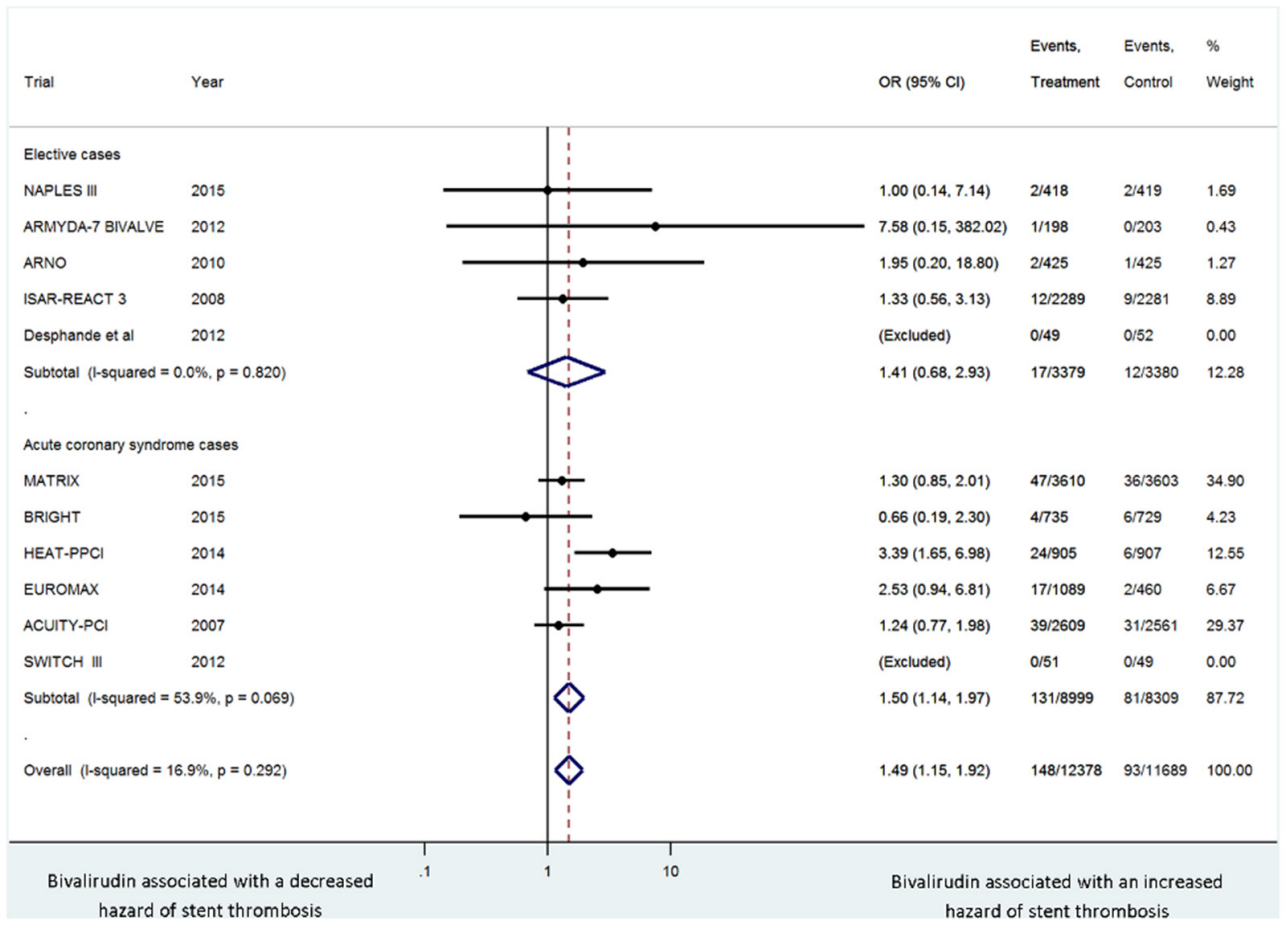
Summary plot of stent thrombosis for bivalirudin versus heparin according to acute coronary syndrome versus elective cases. The relative size of the data markers indicates the weight of the sample size from each study. ACS = acute coronary syndrome; CI = confidence interval; OR = odds ratio.

The incidence of stent thrombosis was 1.2% in the bivalirudin arm versus 0.8% in the heparin arm (OR 1.49, 95% CI 1.15–1.92, *P* = .002, I^2^ = 16.9%) with no evidence of publication bias with Harbord’s test (*P* = .57). This association remained the same in a sensitivity analysis that excluded trials that allowed for upstream use of anticoagulation (MATRIX, SWITCH III, ACUITY-PCI [29,36,42]) (OR 1.96, 95% CI 1.28–3.00, *P* = .002) and in a sensitivity analysis that excluded trials with a modest difference in glycoprotein IIb/IIIa inhibitor usage between study arms (MATRIX, EUROMAX, SWITCH III, ARNO [29,34,36,38]) (OR 1.50, 95% CI 1.07–2.11, *P* = .018). Bivalirudin versus heparin was associated with an increased hazard of acute stent thrombosis (within 24 hours) (OR 2.00, 95% CI 1.23–3.23, *P* = .005), but not with sub-acute stent thrombosis (> 24 hours) (OR 1.07, 95% CI 0.63–1.81, *P* = .80). The excess hazard of stent thrombosis with bivalirudin versus heparin was observed in acute coronary syndrome trials (OR 1.50, 95% CI 1.14–1.97, *P* = .004), but not in elective trials (OR 1.41, 95% CI 0.68–2.93, *P* = .35) (Fig 2).

### Major Bleeding

**Fig 3:**
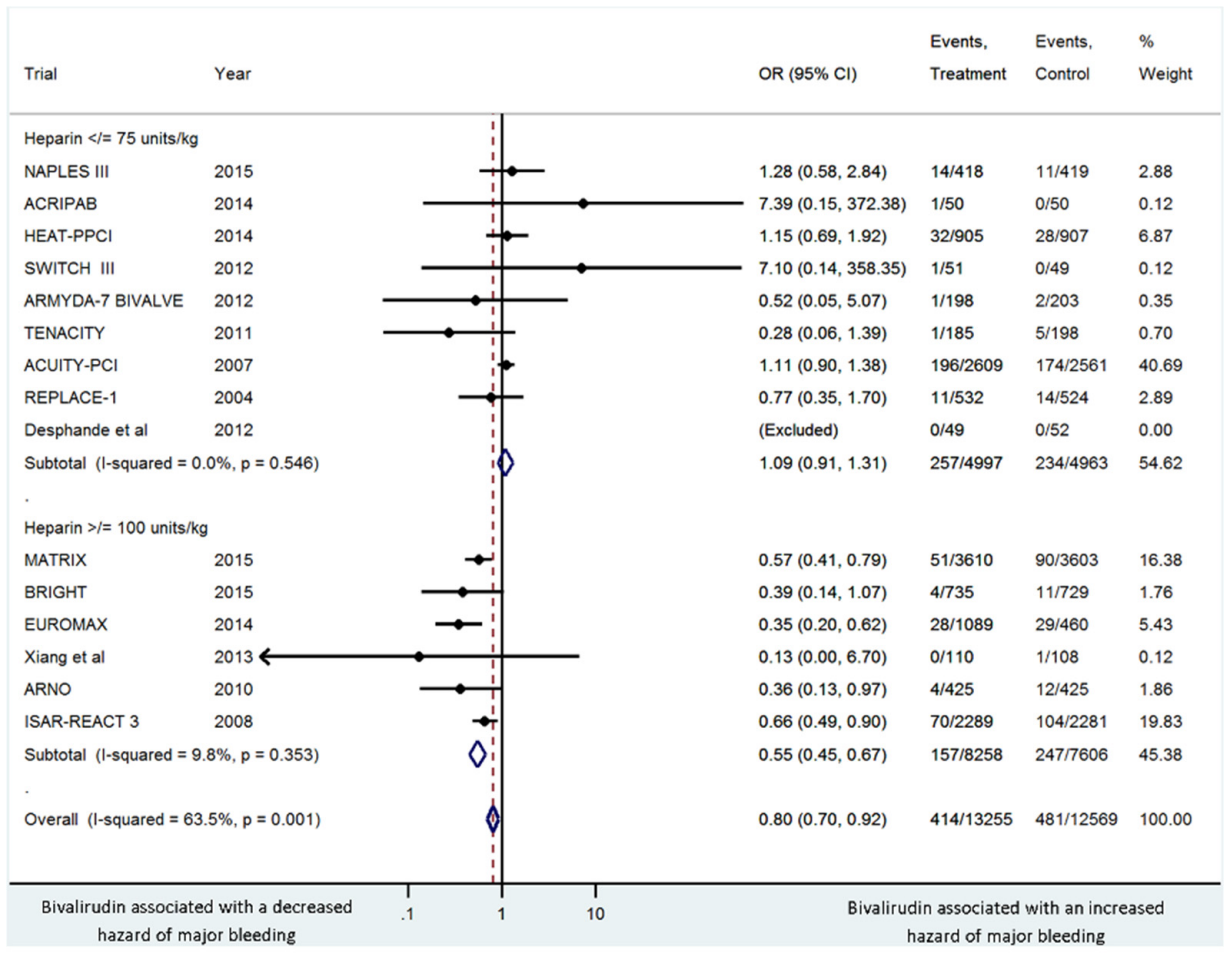
Summary plot of major bleeding for bivalirudin arm versus heparin according to doses ≤ 75 units/kg versus doses ≥ 100 units/kg. The relative size of the data markers indicates the weight of the sample size from each study. CI = confidence interval; OR = odds ratio.

The incidence of major bleeding was 3.1% in the bivalirudin arm versus 3.8% in the heparin arm (OR 0.80, 95% CI 0.70–0.92, *P* = .001, I^2^ = 63.5%), with no evidence of publication bias with Harbord’s test (*P* = .36). This association remained the same in a sensitivity analysis that excluded trials that allowed for upstream use of anticoagulation (MATRIX, SWITCH III, ACUITY-PCI [29,36,42]) (OR 0.66, 95% CI 0.54–0.81, *P* < .0001), but not in a sensitivity analysis that excluded trials with a modest difference in glycoprotein IIb/IIIa inhibitor usage between study arms (MATRIX, EUROMAX, SWITCH III, ARNO [29,34,36,38]) (OR 0.93, 95% CI 0.80–1.08, *P* = .35). The reduced hazard of major bleeding with bivalirudin versus heparin was observed in 1) elective trials (OR 0.69, 95% CI 0.54–0.89, *P* = .005), and in acute coronary syndrome trials (OR 0.85, 95% CI 0.72–0.99, *P* = .04); 2) unfractionated heparin ≥100 units/kg (OR 0.55, 95% CI 0.45–0.68, *P* < .0001), but not with unfractionated heparin ≤ 75 (OR 1.09, 95% CI 0.91–1.31, *P* = .36) (Fig 3); and 3) majority femoral procedures (OR 0.69, 95% CI 0.53–0.89, *P* = .004), and with majority radial procedures (OR 0.61, 95% CI 0.48–0.78, *P* < .0001).

### Other Outcomes

Bivalirudin versus heparin was associated with similar hazard of myocardial infarction (OR 1.09, 95% CI 0.98–1.22, *P* = .11, I^2^ = 35.8%), all-cause mortality (OR 0.88, 95% CI 0.72–1.08, *P* = .21, I^2^ = 31.5%), revascularization (OR 1.23, 95% CI 0.98–1.55, *P* = .077, I^2^ = 27.8%), MACE (OR 1.04, 95% CI 0.94–1.14, *P* = .46, I^2^ = 53.9%), minor bleeding (OR 0.99, 95% CI 0.89–1.10, *P* = .81, I^2^ = 60.6%), and a decreased hazard of net adverse cardiac events (OR 0.91, 95% CI 0.84–0.99, *P* = .028, I^2^ = 67.4%). Outcomes are reported in Table 3.

**Table 3:** Comparison of Summary Estimates for Study Outcomes.

Outcome	Incidence: Bivalirudin, %/UFH, %	Model	Summary estimate (OR)	95% CI	P-value	I^2^%
**Stent Thrombosis**	1.2/0.8	P	1.49	1.15–1.92	0.002	16.9
		SGS	1.83	1.06–3.14	0.033	
**MACE**	7.8/7.6	P	1.04	0.94–1.14	0.46	53.9
		SGS	1.12	0.81–1.55	0.448	
**Mortality**	1.5/1.6	P	0.88	0.72–1.08	0.21	31.5
		SGS	0.76	0.48–1.18	0.200	
**MI**	5.6/5.3	P	1.09	0.98–1.22	0.11	35.8
		SGS	1.18	0.85–1.63	0.309	
**Revascularization**	1.9/1.6	P	1.23	0.98–1.55	0.077	27.8
		SGS	0.86	0.41–1.80	0.65	
**Major bleeding**	3.1/3.8	P	0.80	0.70–0.92	0.001	63.5
		SGS	0.80	0.54–1.18	0.24	
**Minor bleeding**	11.5/11.5	P	0.99	0.89–1.10	0.810	60.6
		SGS	0.94	0.74–1.20	0.591	
**NACE**	10.3/11.3	P	0.91	0.84–0.99	0.028	67.4
		SGS	0.81	0.65–1.00	0.051	

CI = Confidence interval; MACE = major adverse cardiac events; MI = myocardial infarction; NACE = net adverse clinical events; OR = odds ratio; P = Peto method; SGS = Shuster, Guo, and Skyler method; UFH = unfractionated heparin.

## Discussion

Among a broad spectrum of patients undergoing PCI, the use of bivalirudin was associated with a 49% increased hazard for stent thrombosis when compared with heparin. This was due to an increase in acute stent thrombosis. Risk was apparent in acute coronary syndrome trials, but did not achieve significance in elective trials. Stent thrombosis was the most robust finding in this analysis. There was no evidence for publication bias and very little heterogeneity of treatment effect for this outcome. The hazard for stent thrombosis was significantly increased using both the Peto method that is generally favored for outcomes with low event rates [46] and the Shuster, Guo, and Skyler method which was specifically designed for low—event-rate binomial outcomes [47].

The association between bivalirudin and major bleeding was less conclusive. Bivalirudin was associated with a reduction in major bleeding with the Peto method, but not the Shuster, Guo, and Skyler method. This association was no longer significant in a sensitivity analysis that excluded trials with a modest difference in glycoprotein IIb/IIIa inhibitor usage between study arms. There was also significant heterogeneity in major bleeding which required further exploration by sub-group analyses. The only evidence of treatment interaction was with heparin dose. Bivalirudin was only associated with a reduction in major bleeding when compared against high-dose heparin.

Our analysis confirmed the findings of Cassese and others who also concluded that bivalirudin was associated with an increase in stent thrombosis and a reduction in major bleeding compared with heparin [52]. However, that analysis included the entire EUROMAX cohort in which the control arm mostly received heparin plus a routine glycoprotein IIb/IIIa inhibitor [53]. In contrast, we used the pre-specified cohort in which the control arm received heparin plus a bailout glycoprotein IIb/IIIa inhibitor [34]. Compared with that study, our analysis includes 5 additional trials. Bertrand and colleagues concluded that bivalirudin was associated with similar ischemic events and a reduction in major bleeding compared with heparin; however, the majority of their database consisted of observational studies, which are also prone to bias [54]. A strength of our analysis is that it removed the confounding effects of glycoprotein IIb/IIIa inhibitors. In doing so, we revealed that bivalirudin may still be associated with a modest reduction in major bleeding, which was most evident when compared against heparin ≥ 100 units/kg. Therefore, in catheterization laboratories that use heparin doses ≤ 75 units/kg, the use of bivalirudin may not result in a notable reduction in major bleeding.

It is uncertain why bivalirudin was associated with an increased hazard of stent thrombosis when the frequency of ADP antagonists and glycoprotein IIb/IIIa inhibitors was similar between study groups. It is possible that this finding was not an effect of the drug, but rather a process of its use. For example, with unfractionated heparin, activated clotting time (ACT) values are routinely checked and the drug is titrated to achieve therapeutic effect. With bivalirudin, it is generally not recommended to follow ACT values. However, this blood value provides a safety check to ensure that the drug has been infused through a patent peripheral intravenous line and is exerting a systemic effect. In both the Novel Approaches in Preventing or Limiting Event III (NAPLES III) and the How Effective are Antithrombotic Therapies in Primary Percutaneous Coronary Intervention (HEAT-PPCI) trials, which comprised approximately 14% of the study weight of this analysis, ACT values were checked in both study groups [31,33]. Had a peripheral intravenous line become infiltrated in the one of these trials, it would have been discovered and corrected before an adverse event could occur. In distinction, a trial in which ACT values were not checked in the bivalirudin arm was the Acute Catheterization and Urgent Intervention Triage strategy (ACUITY) trial [13]. This trial also allowed for upstream anti-coagulation, which could have resulted in bias by allowing for supra-therapeutic anti-coagulation in the bivalirudin arm. This formed the basis for one of our sensitivity analyses. We found the hazard of stent thrombosis persisted after excluding trials that allowed for upstream anti-coagulation. Accordingly, it is unlikely that lack of uniform ACT evaluation among bivalirudin treated patients could explain our study findings.

Another potential explanation for the increased hazard of stent thrombosis with bivalirudin is that unfractionated heparin has been shown to be a more potent inhibitor of the thrombin-inducible platelet protease-activated receptor (PAR)-1 than bivalirudin. This might result in more potent anti-platelet activity and hence less stent thrombosis with unfractionated heparin [55].

In the Harmonizing Outcomes with Revascularization and Stents in Acute Myocardial Infarction (HORIZONS-AMI) trial, the risk of stent thrombosis was higher with bivalirudin versus unfractionated heparin plus glycoprotein IIb/IIIa inhibitor [9]. The investigators hypothesized this could have been due to the rapid offset of action of bivalirudin prior to maximal anti-platelet effect from ADP antagonists. This influenced the European Ambulance Acute Coronary Syndrome Angiography (EUROMAX) trial design so that bivalirudin was continued at a reduced dose for several hours after the procedure [53]. In addition, most of the patients were treated with either ticagrelor or prasugrel. Despite this design change, there was still an excess hazard of stent thrombosis among bivalirudin treated patients [34,53].

Our study has some limitations. We included trials that compared bivalirudin plus a routine glycoprotein IIb/IIIa inhibitor versus heparin plus a routine glycoprotein IIb/IIIa inhibitor. Although bivalirudin is not routinely used with a glycoprotein IIb/IIIa inhibitor in clinical practice, this randomization was consistent with our study design which eliminated bias from unequal use of glycoprotein IIb/IIIa inhibitors. We included trials that allowed for upstream non-study anti-coagulation (ie, fondaparinux in SWITCH III and heparin in ACUITY). This is also not consistent with clinical practice and may result in bias since ACT values are more routinely measured with heparin; however, our results were materially unchanged in a sensitivity analysis that excluded these trials. In the ARNO trial, all heparin treated patients received protamine following the procedure. This could have caused bias in favor of bivalirudin; however, the findings remained the same after this trial (and MATRIX, EUROMAX and SWITCH III) were excluded in a sensitivity analysis that excluded studies with a modest difference in glycoprotein IIb/IIIa inhibitor usage between study arms. We included the entire spectrum of PCI patients (ie, elective and acute coronary syndromes); however, we considered this approach valid since bivalirudin has been studied and is approved for all of these indications. Lastly, some of the included studies had small sample size, although we included all available studies to avoid any risk of publication bias [56].

## Conclusion

In conclusion, among a broad spectrum of patients undergoing PCI, bivalirudin compared with heparin was associated with an increased hazard for stent thrombosis. This was mostly due to acute stent thrombosis and during acute coronary syndromes. Bivalirudin may be associated with a reduction in major bleeding when compared with unfractionated heparin ≥ 100 units/kg.

## Supporting Information

S1 Table Definition of major bleeding, major adverse cardiac events, and net adverse clinical events among the individual trials. *REPLACE-2 criteria: massive bleeding or life-threatening hemorrhage, such as intracranial hemorrhage, retroperitoneal bleeding, clinically overt bleeding that resulted in a decrease in hemoglobin >3 gram% or transfusion of 2 or more units of packed red blood cells or whole blood. †TIMI criteria: intracranial bleeding or clinically overt bleeding associated with a decrease in hemoglobin >5 g/dl. CABG = coronary artery bypass graft surgery; MI = myocardial infarction; NR = not reported.
(DOCX)

S2 Table Assessment of study quality components. *The clinical events committee was blinded to the treatment allocation. Data are formatted as bivalirudin arm/ unfractionated heparin arm. MI = myocardial infarction; NR = not reported.
(DOCX)

S1 Fig PRISMA checklist. Reports the location in the paper of each item as outlined in the Preferred Reporting Items for Systematic Reviews and Meta-Analyses.
(DOC)
