# Supplementary material for: Critical Appraisal of Bivalirudin versus Heparin for Percutaneous Coronary Intervention: A Meta-Analysis of Randomized Trials
Source: PLoS One. 2015 May 26;10(5):e0127832. doi: 10.1371/journal.pone.0127832 (PMC4444249; doi:10.1371/journal.pone.0127832)
Supplement: S1 Table — *REPLACE-2 criteria: massive bleeding or life-threatening hemorrhage, such as intracranial hemorrhage, retroperitoneal bleeding, clinically overt bleeding that resulted in a decrease in hemoglobin >3 gram% or transfusion of 2 or more units of packed red blood cells or whole blood. †TIMI criteria: intracranial bleeding or clinically overt bleeding associated with a decrease in hemoglobin >5 g/dl. CABG = coronary artery bypass graft surgery; MI = myocardial infarction; NR = not reported. (DOCX) [file pone.0127832.s001.docx]

**S1 Table. Definition of Major Bleeding, Major Adverse Cardiac Events, and Net Adverse Clinical Events Among the Individual Trials.**

| **Trial [ref#]** | **Major bleeding** | | **Major adverse cardiac events** | | **Net adverse clinical events** |
| --- | --- | --- | --- | --- | --- |
| **Bivalirudin plus bail-out glycoprotein IIb/IIIa inhibitor versus heparin plus bail-out glycoprotein IIb/IIIa inhibitor:** | | | | | |
| **MATRIX [29]** | | BARC grade 3 or 5 | | Composite of death, MI, or stroke | Composite of death, MI or stroke or major bleeding |
| **BRIGHT [30]** | | BARC grade 3-5 | | Composite of death, re-infarction, ischemia- driven target vessel revascularization, or stroke | Composite of death, re-infarction, ischemia- driven target vessel revascularization, stroke, or any bleeding |
| **NAPLES III [31]** | | REPLACE-2 criteria^*^ | | Composite of cardiac death, MI, or urgent target vessel revascularization | Composite of cardiac death, MI, urgent target vessel revascularization, or major bleeding |
| **ACRIPAB [32]** | | Intracerebral, intraocular, or retroperitoneal hemorrhage, overt hemoglobin loss > 3 gram%, need for a blood transfusion or for surgical or percutaneous intervention to stop blood loss, or groin hematoma with a circumference > 6 cm | | Composite of cardiac death, target vessel revascularization, stent thrombosis, or any post-PCI ischemic event | NR |
| **HEAT-PPCI [33]** | | BARC grade 3-5 | | Composite of death, re-infarction, unplanned target vessel revascularization, stroke | NR |
| **EUROMAX [34]** | | Intracranial, retroperitoneal, or intraocular bleeding; access-site hemorrhage requiring radiological or surgical intervention; a reduction in the hemoglobin level > 4 gram% without an overt source of bleeding; a reduction in the hemoglobin level > 0.3 gram% with an overt source of bleeding; re-intervention for bleeding; or use of any blood product transfusion | | Composite of death, re-infarction, ischemia-driven revascularization, or stroke | Composite of major adverse cardiovascular events, or non-CABG major bleeding |
| **Xiang et al. [35]** | | REPLACE-2 criteria^*^ | | Composite of death, MI, coronary arterial perforation, pericardial tamponade, no-reflow phenomenon, branch occluding, or acute CABG | NR |
| **SWITCH III [36]** | | Fatal bleeding, symptomatic intracranial hemorrhage, retroperitoneal hemorrhage, intraocular hemorrhage leading to significant vision loss, decrease in hemoglobin ≥3.0 gram%, or bleeding requiring transfusion of ≥ 2 units of red blood cells | | NR | NR |
| **ARMYDA-7 BIVALVE [37]** | | TIMI criteria^†^ | | Composite of cardiac death, MI, target vessel revascularization, or definite or probable stent thrombosis | NR |
| **ARNO [38]** | | REPLACE-2 criteria^*^ | | Composite of death, MI, or unplanned revascularization for ischemia | Composite of death, MI, unplanned revascularization for ischemia, or major bleeding |
| **ISAR-REACT 3 [39]** | | REPLACE-2 criteria^*^ | | Composite of death, MI, or urgent target vessel revascularization | Composite of death, MI, urgent target vessel revascularization or major bleeding |
| **Bivalirudin plus routine glycoprotein IIb/IIIa inhibitor versus heparin plus routine glycoprotein IIb/IIIa inhibitor:** | | | | | |
| **Desphande et al. [40]** | | REPLACE-2 criteria^*^ | | Composite of death, MI, target vessel revascularization for ischemia, or stroke | Composite of death, MI, target vessel revascularization for ischemia, and stroke, or major bleeding |
| **TENACITY [41]** | | REPLACE-2 criteria^*^ and TIMI criteria^†^ | | Composite of death, MI, or urgent target vessel revascularization | Composite of death, MI, urgent target vessel revascularization, or major bleeding |
| **ACUITY-PCI [42]** | | Intracranial or intraocular bleeding, access site hemorrhage requiring intervention, 5 cm or more diameter hematoma, reduction in hemoglobin > 4 gram % or more without or 3 gram % with an overt bleeding source, re-operation for bleeding, or blood product transfusion | | Composite of death, MI, or unplanned revascularization for ischemia | Composite of death, MI, or unplanned revascularization for ischemia, or major bleeding |
| **REPLACE-1 [43]** | | Intracranial, intraocular, or retroperitoneal hemorrhage or clinically overt bleeding resulting in a decrease in hemoglobin by 3 gram% or transfusion of 2 units of blood. | | Composite of death, MI, or repeat revascularization | Composite of death, MI, repeat revascularization, or major bleeding |

^*^REPLACE-2 criteria: massive bleeding or life-threatening hemorrhage, such as intracranial hemorrhage, retroperitoneal bleeding, clinically overt bleeding that resulted in a decrease in hemoglobin >3 gram% or transfusion of 2 or more units of packed red blood cells or whole blood.

^†^TIMI criteria: intracranial bleeding or clinically overt bleeding associated with a decrease in hemoglobin >5 g/dl.

CABG=coronary artery bypass graft surgery; MI= myocardial infarction; NR= not reported.
