# Supplementary material for: Critical Appraisal of Bivalirudin versus Heparin for Percutaneous Coronary Intervention: A Meta-Analysis of Randomized Trials
Source: PLoS One. 2015 May 26;10(5):e0127832. doi: 10.1371/journal.pone.0127832 (PMC4444249; doi:10.1371/journal.pone.0127832)
Supplement: S2 Table — *The clinical events committee was blinded to the treatment allocation. Data are formatted as bivalirudin arm/ unfractionated heparin arm. MI = myocardial infarction; NR = not reported. (DOCX) [file pone.0127832.s002.docx]

**S2 Table. Assessment of Study Quality Components.**

| **Trial (ref#)** | **Primary outcome** | **Single/Multicenter** | **Blinded outcome assessment** | **Generation of treatment assignment** | **Completeness of follow-up, %** |
| --- | --- | --- | --- | --- | --- |
| **Bivalirudin plus a bail-out glycoprotein IIb/IIIa inhibitor versus heparin plus a bail-out glycoprotein IIb/IIIa inhibitor:** | | | | | |
| **MATRIX [29]** | Composite of death, MI and stroke/ Composite of death, MI or stroke, or major bleeding | Multicenter | Open label^*^ | NR | NR |
| **BRIGHT [30]** | Composite of death, re-infarction, revascularization, stroke, or any bleeding | Multicenter | Open label^*^ | Sealed envelope | 100/100 |
| **NAPLES III [31]** | Major bleeding | Single center | Double blinded, investigator initiated | NR | 100/100 |
| **ACRIPAB [32]** | Major and minor bleeding, or entry site complication | Single center | Double blinded | Opaque, sealed envelope | 100/100 |
| **HEAT-PPCI [33]** | Composite of death, stroke, re-infarction, and revascularization, or major bleeding | Single center | Open label^*^ | Computer-generated | 100/99.8 |
| **EUROMAX [34]** | Composite of death or major bleeding | Multicenter | Open label^*^ | Opaque, sealed envelope/central randomization system | 100/100 |
| **Xiang et al. [35]** | Procedure success rate and bleeding | Multicenter | Single blinded | Random assignment | 95.5/88.9 |
| **SWITCH III [36]** | Major bleeding | Multicenter | Open label | Computer-generated | 100/100 |
| **ARMYDA-7 BIVALVE [37]** | Composite of cardiac death, MI, revascularization, stent thrombosis or any bleeding event, or entry site complication | 2 centers | Open label | Random numbers | 100/100 |
| **ARNO [38]** | Major bleeding | Single center | Open label^*^ | Computer-generated | 100/100 |
| **ISAR-REACT 3 [39]** | Composite of death, MI, revascularization, or major bleeding | Multicenter | Double blinded | Opaque, sealed envelope | 100/100 |
| **Bivalirudin plus a routine glycoprotein IIb/IIIa inhibitor versus heparin plus a routine glycoprotein IIb/IIIa inhibitor:** | | | | | |
| **Desphande et al. [40]** | Post procedural time to sheath removal | Single center | Open label | Random assignment | 100/100 |
| **TENACITY [41]** | Composite of death, MI, or revascularization | Multicenter | Double blinded | NR | NR |
| **ACUITY-PCI [42]** | Composite of death, MI, revascularization or major bleeding or the combination of both | Multicenter | Open label^*^ | Random numbers | 99.7/99.8 |
| **REPLACE-1 [43]** | Composite of death, MI, or revascularization | Multicenter | Open label | Central telephone system | 100/100 |

^*^ The clinical events committee was blinded to the treatment allocation.

Data are formatted as bivalirudin arm/ unfractionated heparin arm.

MI = myocardial infarction; NR = not reported.
